# The Relationship between Burnout and Wellbeing Using Social Support, Organizational Justice, and Lifelong Learning in Healthcare Specialists from Romania

**DOI:** 10.3390/medicina59071352

**Published:** 2023-07-24

**Authors:** Roxana Mihaela Claponea, Magdalena Iorga

**Affiliations:** 1 Faculty of Psychology and Education Sciences, “Alexandru Ioan Cuza” University of Iasi, 700506 Iasi, Romania; roxana.claponea@student.uaic.ro; 2 Faculty of Medicine, “Grigore T. Popa” University of Medicine and Pharmacy Iasi, 700115 Iasi, Romania

**Keywords:** medical staff, non-medical staff, burnout, organizational justice, wellbeing, social support, lifelong learning

## Abstract

*Background and objectives*: The goal of this study was to evaluate the levels of organizational justice, social support, wellbeing, and lifelong learning associated with the level of burnout experienced by medical and non-medical staff from public and private medical units. *Materials and Methods*: A cross-sectional study was conducted on a sample of 497 healthcare professionals: 367 medical personnel (M_age_ = 43.75 ± 0.50), including 216 nurses, 97 physicians, and 54 respondents with other medical specialities such as biologists, psychologists, physical therapists, pharmacists, etc., and 130 non-medical staff respondents (M_age_ = 45.63 ± 0.80), including administrative personnel. The Maslach Burnout Inventory, the ECO System, the Multidimensional Scale of Perceived Social Support, the WHO Wellbeing Index, and the revised Jefferson Scale of Physician’s Lifelong Learning were used. *Results*: Burnout was measured in terms of emotional exhaustion, depersonalization, and personal accomplishment. Medical personnel registered higher values of personal accomplishment (38.66 ± 0.39 vs. 35.87 ± 0.69), while non-medical personnel registered higher values of depersonalization (6.59 ± 0.52 vs. 4.43 ± 0.26) and emotional exhaustion (27.33 ± 1.24 vs. 19.67 ± 0.71). In terms of organizational justice, higher scores were observed for medical staff, while non-medical staff recorded lower values (24.28 ± 0.24 vs. 22.14 ± 0.38). For wellbeing, higher scores were also registered for medical staff (11.95 ± 0.21 vs. 10.33 ± 0.37). *Conclusions*: For lifelong learning and social support, no statistically significant differences were found. In the case of the proposed parallel moderated mediation model, the moderated mediation effects of organizational justice, lifelong learning, and burnout on the relationship between social support and wellbeing were valid for every dimension of burnout (emotional exhaustion, depersonalization, and personal accomplishment), but lifelong learning was not found to be a viable mediating variable, even if high levels of social support correspond to high levels of lifelong learning and wellbeing.

## 1. Introduction

Usually, in a healthcare unit, there are two different categories of personnel: medical personnel and non-medical personnel. The medical staff in hospitals consists of a variety of professionals who work together to provide care to patients. Some of the most important members of the medical staff in hospitals are physicians, nurses, physical therapists, chemists, biologists, pharmacists, social workers, and psychologists. In general, the medical staff in hospitals consists of a team of dedicated and well-trained professionals who work together to provide care for patients and ensure their safety and wellbeing. On the other hand, in general, administrative personnel are represented by economists, legal advisers, human resources specialists, auditors, etc. Administrative staff in hospitals have an important role in ensuring the smooth functioning of the institution and ensuring that all activities are carried out efficiently and in accordance with legal standards and institutional guidelines.

The initial study of burnout appeared in the mid-1970s in an article published by Freudenberger, which defined burnout as the process by which an individual experiences emotional depletion and a loss of commitment or motivation [1]. Burnout is defined as a syndrome consisting of three different stages: emotional exhaustion, depersonalization, and reduced feelings of personal accomplishment [2]. Burnout is a syndrome of nervous exhaustion caused by overwork that depletes the nervous system’s emotional and physical functioning capacity. Employees have a limited capacity for information absorption and, through overwork, reach states of fatigue and exhaustion that result in diminished work performance [3]. Long-term exposure to work-related stressors can lead to burnout. Maslach and Jackson (1981) defined this as the experience of emotional exhaustion (the depletion or drain of one’s emotional resources), depersonalization (the development of negative, unemphatic, and cynical attitudes toward patients), and the reduction in personal accomplishments (the tendency to value work-related behaviour and performance in a negative way) [4]. Factors at work that can cause somatic problems, stress, or burnout are represented by organizational psychosocial risks. Among the most important predictors of the appearance of psychosomatic symptoms as potential psychosocial risk factors are: overload at work, lack of organizational justice, professional (non-)satisfaction, and the leadership style of direct superiors [5]. In the same sense, adequate management of psychosocial risks at work helps prevent accidents and absenteeism [6] and increases wellbeing [7].

Organizational justice indicates the level of fairness in the decision-making process, the procedures used in taking decisions, and the interpersonal treatment of personnel [8]. Hence, organizational justice takes into consideration the perception of fairness at the organizational level [9]. All of these refer to promotion, payment, recognition, and job resources that the employee needs in order to accomplish his or her tasks [10]. Organizational justice refers to how an organization treats its employees regarding justice issues. This may include policies and practices that promote fair and non-discriminatory treatment, protection against harassment and other unwanted behaviour, and fostering a culture of transparency and accountability. Previous studies suggest that in the case of healthcare personnel, reducing overload and promoting organizational justice and professional identification are key factors that could be addressed simultaneously and independently in interventions to reduce the risk of burnout or prevent it [11].

Wellbeing takes a holistic view that considers the physical, mental, and emotional aspects of an employee’s health. Therefore, wellbeing considers health, the life experiences of the employee, and experiences in the organizational context [12]. Findings on wellbeing and coping strategies for stress and burnout suggest that positive coping strategies (planning, acceptance, positive reframing, and social support) are positively associated with wellbeing and quality of life [13]. A work environment designed to support the positive development of energy, vigour, engagement, dedication, absorption, and effectiveness among employees should be successful in promoting wellbeing [14].

Social support has been considered an alternative method of self-care that consists of developing supportive relationships. These involve a manageable schedule that balances the needs of others (school, work, and family) with what is imperative for the individual. Alternative features of the support structure relate to the physical atmosphere. This includes maintaining a comfortable living environment and a controlled work area [15]. Quality of life is correlated with achieving a healthy and productive lifestyle. The WHO defines quality of life as an individual’s perception of their position in life in the context of the culture and value systems in which they live and in relation to their goals, expectations, standards, and concerns. It is a broad concept affected in a complex way by the individual’s physical health, psychological state, level of independence, social relationships, beliefs, and their relationship with the essential features of their environment [16]. At the same time, it is indicated that receiving social support outside of work through family, friends, and colleagues can act positively in preventing burnout syndrome. Social support refers to social interaction in which supportive resources are received from others. Social support helps manage uncertainty, increases the perception of personal control over life experiences, and helps achieve goals [17].

Another important aspect for healthcare personnel is lifelong learning. To stay abreast of new evidence and procedures that are being developed, healthcare professionals must engage in continuing professional development. The lack of work–life satisfaction has been correlated with emotional exhaustion [18,19] and can harm the doctor-patient relationship, leading to unsatisfactory treatment outcomes [20]. Thus, health professionals must choose to learn throughout their professional careers, spending time to keep up with advances in their specialty. This task becomes increasingly difficult in a society where scientific, technological, and social changes occur rapidly. Lifelong learning is an active process in which the individuals seek knowledge and understanding and use them to meet their professional needs throughout their lives. Lifelong learning includes formal and informal learning and learner independence (self-direction), which is one of the major characteristics of lifelong learning [21].

Burnout has significant negative effects on an individual’s overall wellbeing. It negatively impacts various aspects of a person’s health, including their physical, mental, and emotional wellbeing, ultimately reducing their overall quality of life.

Burnout appears to be associated with multiple self-reported indicators of personal distress, such as feelings of physical exhaustion, difficulty sleeping, heightened substance use, and challenges within marital and family relationships [4]. Burnout may affect mental health because individuals may struggle with cognitive difficulties, including diminished concentration, memory problems, and decreased productivity. Moreover, individuals may experience a loss of interest and engagement in previously enjoyed activities, further diminishing their overall psychological wellbeing [18,19].

Burnout can also erode emotional wellbeing, making individuals more vulnerable to emotional distress. Feelings of irritability, cynicism, and detachment are common in individuals experiencing burnout. Burnout can impair emotional regulation, leading to emotional exhaustion and a failure to effectively cope with stressors because positive relationships and social support are crucial in managing employee wellbeing [13].

Ultimately, burnout takes a toll on an individual’s overall quality of life. The combination of physical, mental, and emotional challenges associated with burnout can lead to a reduced sense of purpose, personal accomplishment, and enjoyment in various life domains. It can also strain personal relationships, as individuals may have limited energy and emotional resources to devote to their social connections and leisure activities. On the whole, burnout diminishes the overall quality of life and prevents individuals from fully engaging and thriving in their personal and professional lives [12,14].

The present study aims to analyse the differences between the two categories of staff in health facilities, medical staff and non-medical staff, in terms of burnout felt, social support, wellbeing, lifelong learning, and organizational justice. To test a unique and predictive model, we analysed a moderated mediation pattern of organisational justice, lifelong learning, and burnout on the relationship between social support and wellbeing. The hypotheses formulated for this research are as follows:

**H1.**  *There are significant differences between medical and non-medical staff regarding all three burnout subscales, organizational justice, wellbeing, social support, and lifelong learning.*

Research has shown that progress from low burnout to high burnout was correlated with a worsening of personal wellbeing or workplace wellbeing indicators [2]. Even prior to COVID-19, the wellbeing of doctors was described internationally in terms of burnout, with 32% to 80% of surveyed doctors being at high risk of “burnout” [22]. However, “professional burnout” is a pathological condition described in the International Classification of Diseases [23], whereas “wellbeing” is a positive noun. We measure wellbeing as if it were a pathology that limits ambitions to survival rather than becoming healthy, without the ability to measure prosperity. Thus, higher wellbeing experienced by medical staff could be evidenced by feelings of rigour, willingness, and personal and professional activities undertaken out of passion.

**H2.**  *Lifelong learning and burnout will mediate the relationship between social support and wellbeing, while organizational justice will moderate the relationship between the mediators and the criterion variable.*

**H2a.**  *Lifelong learning and emotional exhaustion will mediate the relationship between social support and wellbeing, while organizational justice will moderate the relationship between the mediators and the criterion variable.*

**H2b.**  *Personal accomplishment will mediate the relationship between social support and wellbeing, while organizational justice will moderate the relationship between the mediator and the criterion variable.*

**H2c.**  *Depersonalization will mediate the relationship between social support and wellbeing, while organizational justice will moderate the relationship between the mediator and the criterion variable.*

Lifelong learning is a complex concept, as reflected in the following definition: “Lifelong learning is the development of human potential through a continuous process of support that stimulates and empowers individuals to acquire all the knowledge, values, skills, and understanding they will need throughout their lives, with confidence, creativity, and enjoyment in all roles, circumstances, and environments” [24]. Other authors also show a serial mediation in which shared values are related to perceptions of organizational justice, which in turn have been negatively correlated with emotional exhaustion that subsequently relates to higher levels of workplace wellbeing [25]. In terms of social support and wellbeing, it has been suggested that when an individual perceives greater autonomy support from their friend, the individual also provides more autonomy support to the friend. Receiving support also predicted a higher level of the recipient’s psychological wellbeing [26].

## 2. Materials and Methods

### 2.1. Study Design and Population

From February–March 2023, a cross-sectional study was carried out using the snowball sampling method. A questionnaire was created and distributed online using Google Forms. The questionnaire was distributed with the help of the Order of General Medical Assistants, Midwives, and Medical Assistants from Romania and heads of departments from public and private medical units from Iasi, Constanta, Oradea, Cluj, Bucharest, and Timis. Furthermore, the questionnaire was distributed via the Pharmacists College from Romania, the Psychologists College from Constanta, the Association of Legal Advisors from Public Hospitals, the Association of Quality Management Officers in Public Hospitals, and Sanitas Constanta Trade Union.

The snowball sampling method is a non-probability sampling technique commonly used in research to identify and recruit participants. It involves initially selecting a small number of individuals who meet the required criteria and then asking them to provide referrals to other potential participants who also meet the criteria. This process creates a “snowball effect” as the sample size grows. Firstly, an initial set of participants was identified and selected based on their knowledge on the research topic. Therefore, the questionnaire was addressed to participants from the main Romanian professional organizations in the healthcare system. This process allowed the sample size to gradually increase. The analysis continued with the monitoring of the sample characteristics to ensure diversity and representation, tracking demographic information such as age, gender, speciality, and type of medical unit. The process ended with data saturation. Hence, the snowball sampling process continued until data saturation was reached. Data saturation occurred when additional participants no longer provided substantially new or different information relevant to the research objectives. In the end, the rate of response was 62%, since 497 responses were collected.

The group of participants consisted of 367 medical staff respondents (doctors, nurses, physical therapists, biologists, pharmacists, psychologists, social workers, etc.) and 130 non-medical (administrative) staff respondents who worked in hospitals and health facilities in Romania. The participants worked in municipal, clinical, regional, county, or polycyclic hospitals in both the private and public sectors.

Participants were included if they were currently employed in Romanian hospitals/clinics/state/private institutions. Participants who submitted their answers after the deadline were excluded.

The respondents were divided into two groups based on their professions to understand sector-specific challenges. Medical and non-medical personnel often handle distinct challenges within their relevant fields, and sector-specific factors contributing to burnout could be identified. Furthermore, comparisons between these different categories of personnel are lacking in the literature, with non-medical personnel being rarely investigated in healthcare studies, especially in Romania. Analysing medical and non-medical personnel provided an opportunity to compare their experiences. This comparative analysis highlighted the differences in symptoms and the impact of burnout across different fields. Previous studies revealed that non-medical personnel faced higher levels of workload, emotional exhaustion, and depersonalization, and lower scores for personal accomplishment [4,27]. Hence, we considered the positive variables associated with burnout in healthcare professionals from Romania, such as social support, wellbeing, and lifelong learning. Therefore, dividing participants into sub-samples based on their field of activity identified specific characteristics, experiences, and perspectives of the two groups that have totally different duties. While medical and non-medical personnel have distinct roles, they often interact and collaborate within healthcare situations. While there are benefits to conducting research on medical and non-medical personnel separately, it is also important to consider the advantages of analysing them together, encouraging a comprehensive understanding of burnout across the entire sample.

The informed consent provided data on the purpose of the study and possibility to abandon the questionnaire with no consequence. No bonuses were offered to the respondents. The participation was voluntary and anonymous. Continuing to complete the questionnaire implied their consent to participate in the study.

### 2.2. Study Instruments

The questionnaire was constructed using the Google Forms application (Alphabet, Mountain View, CA, USA).

(a) Socio-demographic, medical, professional, and institutional data were collected,
(b) Psychological instruments were used in order to measure the level of burnout, organizational justice, lifelong learning, social support, and wellbeing.

Burnout: The 22-item Maslach Burnout Inventory-Human Services Survey (MBI-HSS) measuring instrument [4,27] was applied, being one of the most widely used variants of the tool. The instrument contains 22 items, with answers on a Likert scale from 0 (never) to 6 (always). The instrument evaluated the three dimensions of burnout. Examples of items are: *I feel emotionally drained from my work* (emotional exhaustion—EE); *I feel very energetic* (personal accomplishment—PA); and *I’ve become more callous towards people since I took this job* (depersonalization—D). High scores for the EE and D dimensions and low scores for PA correspond to higher levels of burnout [28]. In the research sample, the Cronbach’s alpha consistency coefficients for each subscale range from acceptable to excellent, namely α = 0.94 (EE), α = 0.79 (PA), and α = 0.70 (D).

Organizational justice: The ECO system [29] was used for measuring organizational justice via the dimension of organizational justice (8 items) to measure the psychosocial risks at the organizational level using a 5-point Likert scale, ranging from 1 = to a very small extent to 5 = to a very large extent. An example of items is: *The salary I receive is correct relative to the work I am doing*. This instrument was used where employees had identical professional pressures and working conditions but held different positions in an organization. This dimension registered adequate psychometric properties in previous studies (Cronbach’s alpha > 0.81) [5]. For the present study we obtained an α = 0.70.

Wellbeing: The WHO Wellbeing Index (WHO-5) [23] was used to measure wellbeing according to five criteria on a six-point Likert scale (0 = at no time, 5 = all of the time). An example criterion is “*I have felt calm and relaxed*”. For the present study, we obtained an α = 0.90.

Social Support: The Multidimensional Scale of Perceived Social Support (MSPSS)—[30] was used. The scale contains 12 items and is used to evaluate social support from the perspectives of family, friends, and significant others. The answers ranged from 1 = very strongly disagree to 7 = very strongly agree, with a higher score predicting greater perceived social support and a low score describing a low level of social support. An example item is “*There is a special person who is around when I am in need*”. We obtained an α = 0.93 for the present research.

Lifelong learning: The Revised Jefferson Scale of Physician’s Lifelong Learning (JeffSPLL) was used. The JeffSPLL measures a health professional’s ability to seek information, self-motivate, and capitalize on learning opportunities [31]. The 14 items on the JeffSPLL evaluate three factors: beliefs and motivations in learning, attention to learning opportunities, and information seeking skills, with answers on a four-point Likert scale, ranging from 1 = strongly disagree to 4 = strongly agree (e.g., *I believe that I would fall behind if I stopped learning about new developments in my profession*). Higher scores were associated with greater development of the measured items. We obtained an α = 0.91 for the lifelong learning scale.

### 2.3. Statistical Analysis

The IBM Statistical Package for Social Sciences (SPSS) and Statistics for Windows, version 29 (SPSS Inc., Chicago, IL, USA) were used to test the hypotheses [32] and to perform the moderated mediation analysis. The first group included medical personnel (doctor, nurse, physical therapist, psychologist, pharmacist, biologist, chemist, and social worker) and the second one included non-medical personnel (administrative staff). In the second part of the analysis, medical and non-medical personnel were analysed together.

### 2.4. Ethical Statement

The study was conducted in accordance with the Declaration of Helsinki and approved by the Institutional Board of the Association of Legal Advisors from Public Hospitals in Romania, Research Ethical Agreement No. 54/15, September 2022.

## 3. Results

### 3.1. Socio-Demographic and Job-Related Data

The sample consisted of 367 medical staff respondents and 130 administrative staff respondents. Demographic data are presented in Table 1. The majority of participants were women (85.11%), with a mean age of 43.75 for medical staff (M = 43.75, ±0.50) and 45.63 (M = 45.63, ±0.80) for non-medical staff; 80.28% were in a relationship; 75.86% had children; 41.45% had more than 20 years of work experience; 72.43% worked in a public institution and 35.01% in an emergency unit; 24.55% had a management position; 37.63% had a bachelor’s degree; and 29.58% suffered from chronic diseases.

### 3.2. Descriptive Statistics

The descriptive data of the variables of interest, together with the Pearson correlation coefficients between them, can be found in Table 2. It can be seen already from the descriptive data that the average of the medical staff falls into the category of moderate emotional exhaustion (17–26 points), while the average of the administrative staff falls into the category of high emotional exhaustion (27+ points), while both groups fall into the low category of depersonalization and the moderate category of personal accomplishment, according to the test manual [33]. From the point of view of social support, both groups have high social support, according to the test manual [30].

Strong correlations can be observed between the variables of interest for both groups, such as the negative correlation between wellbeing and emotional exhaustion or that between organizational justice and emotional exhaustion, the latter being stronger in the case of medical staff.

### 3.3. Inferential Data Analysis

**H1.**  *There are significant differences between medical and non-medical staff regarding emotional exhaustion, personal accomplishment, depersonalization, organizational justice, wellbeing, social support, and lifelong learning.*

As can be seen in Table 3, significant differences were found between the two groups in the case of burnout dimensions (emotional exhaustion, personal accomplishment, and depersonalization), but also in the case of organizational justice and wellbeing. Therefore, the results indicate that administrative staff have, on average, a statistically significantly higher score in EE and D, while medical staff have a statistically significantly higher score in PA, organizational justice, and wellbeing. Medical personnel registered higher values of PA (38.66 ± 0.39 vs. 35.87 ± 0.69), while non-medical personnel registered higher values of D (6.59 ± 0.52 vs. 4.43 ± 0.26), and EE (27.33 ± 1.24 vs. 19.67 ± 0.71). For organizational justice, higher scores were observed for medical staff, while non-medical staff recorded lower values (24.28 ± 0.24 vs. 22.14 ± 0.38). In terms of wellbeing, higher scores were also registered for medical staff (11.95 ± 0.21 vs. 10.33 ± 0.37). In the case of lifelong learning and social support, no statistically significant differences were found.

**H2.**  *Lifelong learning and burnout will mediate the relationship between social support and wellbeing, while organizational justice will moderate the relationship between the mediators and the criterion variable*.

To test this hypothesis, a parallel mediation model was applied; the social support variable was the predictor variable, the mediating variables were lifelong learning and, in turn, each dimension of burnout (EE, PA, and D), and the moderator variable was organizational justice. Thus, the following hypotheses are issued:

**H2a.**  *Lifelong learning and emotional exhaustion will mediate the relationship between social support and wellbeing, while organizational justice will moderate the relationship between the mediators and the criterion variable.*

**Figure 1:**
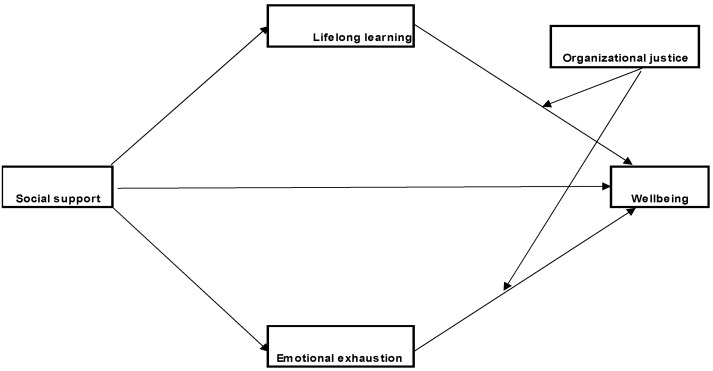
Model 1. The designed parallel moderated mediation model.

The first model tested (Figure 1, Model 1) represents the moderated mediating effect of organizational justice, lifelong learning, and emotional exhaustion on the relationship between social support and wellbeing.

**Figure 2:**
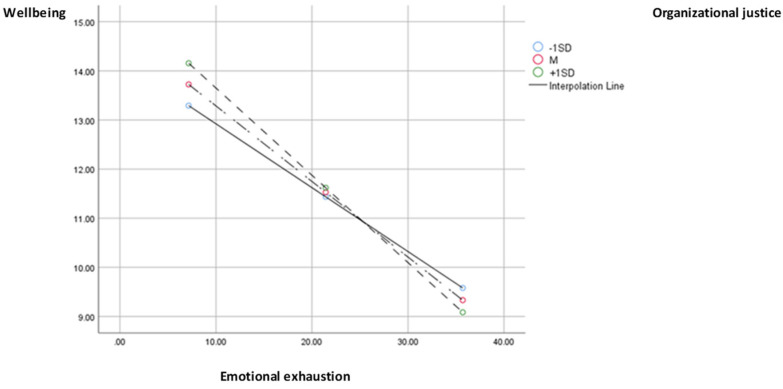
Chart of the moderated effect of organizational justice through emotional exhaustion on wellbeing.

The results of the moderated mediation analysis indicate that social support has a statistically significant direct effect on emotional exhaustion (path a2; b = −2.43, SE = 0.63, *p* < 0.001), lifelong learning (path a1; b = 1.18, SE = 0.27, *p* < 0.001), and wellbeing (path c’; b = 0.97, SE = 0.16, *p* < 0.001). The effect on EE is negative, indicating that a high level of social support refers to a low level of emotional exhaustion. However, the effect on lifelong learning and wellbeing is positive, i.e., high social support corresponds to high levels of lifelong learning and wellbeing. However, among the interaction effects of organizational justice on the two mediators, only the interaction with emotional exhaustion is significant (b = −0.005, SE = 0.00, *p* < 0.05), indicating the existence of considerable moderation. Finally, mediation is significant only through emotional exhaustion (path a2b2), at high levels of the moderator (+1SD; b = 0.43, SE = 0.12, 95% CI [0.19, 0.67]) and at medium (M; b = 0.37, SE = 0.10, 95% CI [0.17, 0.57]) and low levels (−1SD; b = 0.31, SE = 0.09, 95% CI [0.14, 0.49]), the indirect effect being positive in all 3 cases (Figure 2). The index of moderated mediation indicates that the results are substantial (I = 0.01, SE = 0.00, 95% CI [0.006, 0.02

**H2b.**  *Personal accomplishment will mediate the relationship between social support and wellbeing, while organizational justice will moderate the relationship between the mediator and the criterion variable.*

**Figure 3:**
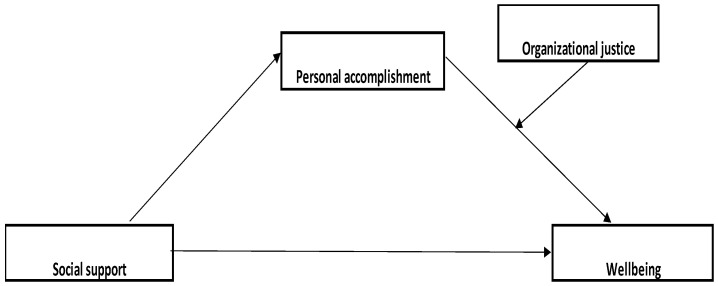
Model 2. The designed parallel moderated mediation model.

Since the testing of the first model of moderate mediation revealed that both the indirect effect through lifelong learning and the interaction between lifelong learning and organizational justice were insignificant, the lifelong learning variable was removed from this model. As can be seen in Figure 3, Model 2, the same model of moderate mediation is applied, but with the positive dimension of burnout, namely personal accomplishment. Like the first model, it is observed that social support has a significant effect on personal accomplishment (b = 2.09, SE = 0.34, *p* < 0.001), this time being a positive relationship, which means that when there is high social support, personal accomplishment is also high.

**Figure 4:**
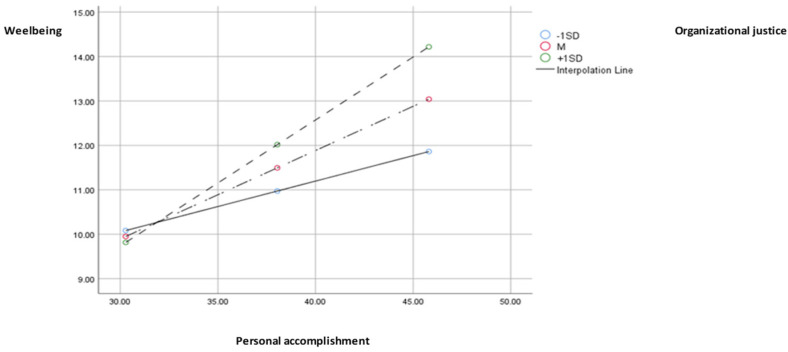
Chart of the moderated effect of organizational justice through personal accomplishment on wellbeing.

There is also a significant effect of personal accomplishment on wellbeing (b = −0.22, SE = 0.10, *p* < 0.05), and the interaction effect between organizational justice and personal accomplishment is also significant (b = 0.01)., SE = 0.00, *p* < 0.001). The effect of personal accomplishment on wellbeing (Figure 4) is significant and positive at small values (−1SD) of the moderator (b = 0.11, SE = 0.02, 95% CI [0.06, 0.16]), as well as at mean (M; b = 0.43, SE = 0.19, 95% CI [0.15, 0.24]) and high values (+1SD; b = 0.28, SE = 0.03, 95% CI [0.21, 0.35]). However, the Johnson-Neyman region of significance indicates that at very low values of organizational justice (5th percentile), the effect of personal accomplishment becomes insignificant (b = 0.06, SE = 0.03, 95% CI [−0.002; 0.13]). The indirect effect of social support, through personal accomplishment and interaction with organizational justice, is also positive for small (−1SD), medium (M), or large (+1SD) values of the moderator, and the moderated mediation index indicates that a moderately significant mediation (I = 0.03, SE = 0.01, 95% CI [0.01, 0.06]) exists. The model explains 26% of the variance in wellbeing.

**H2c.**  *Depersonalization will mediate the relationship between social support and wellbeing, while organizational justice will moderate the relationship between the mediator and the criterion variable.*

**Figure 5:**
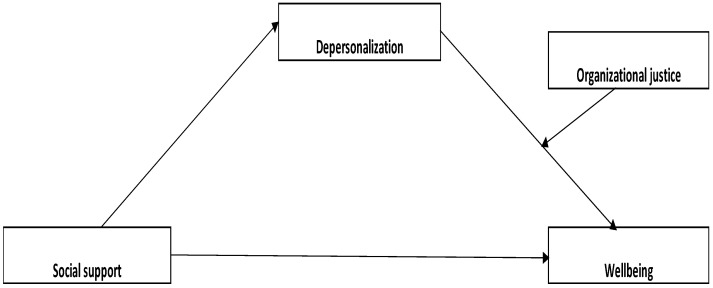
Model 3. The designed parallel moderated mediation model.

**Figure 6:**
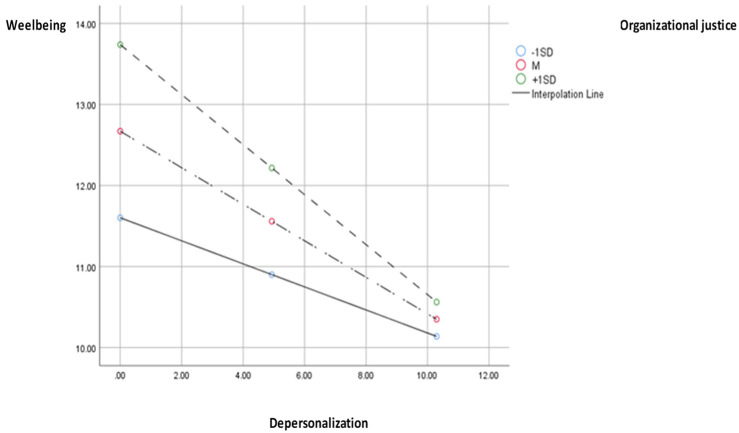
Chart of the moderated effect of organizational justice through depersonalization on wellbeing.

As we can see in Figure 5, Model 3, the analysis of data reflected that social support has a significant effect on depersonalization (b = −0.70, SE = 0.24, *p* < 0.01), and the interaction between depersonalization and wellbeing is also significant (b = −0.01, SE = 0.00, *p* < 0.05). The effects of depersonalization on wellbeing are significant and negative, at small (−1SD; b = −0.14, SE = 0.04, *p* < 0.001), medium (M; b = −0.22, SE = 0.03), *p* < 0.001), and large (+1SD; b = −0.30, SE = 0.05, *p* < 0.001) levels of the moderator (Figure 6). However, the effect of depersonalization on wellbeing becomes insignificant at extremely low values of organizational justice (8th percentile). The indirect effect of social support through depersonalization, moderated by organizational justice, is notable and positive, and the moderated mediation index is also significant (I = 0.01, SE = 0.00, 95% CI [0.009; 0.02]). The model is considerable and explains 22% of the variance in wellbeing.

## 4. Discussion

The administrative staff appears to experience higher levels of emotional exhaustion and depersonalization, which can be associated with symptoms such as fatigue, a lack of motivation, and a cynical attitude towards stakeholders or colleagues. These negative effects can be amplified by high workloads, limited resources, and workplace stress. On the other hand, medical personnel have a significantly higher score in terms of personal accomplishment, organizational justice, and wellbeing. These can be associated with the satisfaction of helping people, a sense of belonging to a team, and respect and gratitude from patients and the community. Medical and administrative staff in the healthcare system have different roles in providing healthcare services and may therefore experience differences in terms of social support, burnout, and wellbeing. Regarding burnout, doctors and nurses are often more susceptible to it due to the stressful and demanding nature of their work as well as the high responsibility they have in caring for patients. In contrast, administrative staff may experience burnout due to excessive pressure from managers, overwork, constant rule and policy changes, financial stress, or other organizational issues. Regarding wellbeing, medical staff may enjoy a greater sense of satisfaction and personal accomplishment because they can see the results of their work at the level of the patients they care for. In contrast, administrative staff may experience less professional satisfaction and fulfilment due to the administrative nature of their work, which does not offer the same emotional and psychological satisfaction as direct medical care.

In general, the perception of organizational justice can be influenced by several factors, such as the organization’s policies and procedures, how employees are treated, communication in the organization, and the level of employee participation and involvement in decision-making processes. Medical personnel experience a higher level of organizational justice and personal accomplishment than non-medical personnel. These results are similar to previous research that revealed a higher level in the case of these dimensions for medical personnel [34]. A higher wellbeing score for medical staff was also recorded. In the case of EE, high scores were revealed for non-medical staff and average scores were revealed for medical staff. Regarding the EE dimension, similar results were highlighted in previous studies among medical professionals [35]. In the case of the D dimension, low scores were recorded for the medical staff and for the administrative staff. Low scores for the D dimension were previously found in the case of medical professionals, represented by nurses [36,37]. Another study that analysed the level of burnout among specialized staff in the medical sector, including administrative staff, reflected low scores for the D dimension, suggesting workplace optimism as the source of low scores for this dimension [38]. In the case of the depersonalization dimension, low scores were recorded for both categories of staff, but scores were higher in the case of non-medical staff, highlighting a higher degree of detachment in relation to the professional activity of administrative staff. PA registered average values in the case of both categories of staff analysed, similar scores being recorded in other studies that analysed the level of burnout among health care staff [36]. Thus, although administrative staff receive high scores in EE, they receive moderate scores in the personal accomplishment dimension and low scores in the D dimension. Thus, EE may originate from feelings of frustration at work, pressure from others at work, or emotional dryness.

However, these considerations do not lead to low personal accomplishment but to moderate scores, which may be derived from successful task solving, positive influence on those with whom they interact at work, or professional achievements at work. In terms of moderate levels of the personal accomplishment dimension, similar scores have been recorded for health specialists [38]. In the case of medical staff, emotional exhaustion scores lower, as does depersonalization, which does not necessarily lead to high personal accomplishment scores but does result in moderate overall scores and significantly higher scores than for administrative staff. Personal accomplishment may stem from patient-related work or workplace achievements, whether it is saving a patient in the case of a doctor or reducing pain in the case of medical rehabilitation procedures performed by a nurse or physiotherapist. There is also a higher level of organizational justice felt by medical staff—one determined by the inequities in salaries created among medical and administrative staff, against the backdrop of a busy work schedule filled with constant legislative changes that can also create organizational controversy. We could have assessed that this injustice could also be caused by salary inequalities between the two categories of staff, since unlike medical staff, who benefited from a substantially higher salary income from 2018 onwards, according to the Law on the Salary of Staff Paid from Public Funds, number 153/2017, administrative staff did not benefit from the salary increases imposed by the law in force, being constantly subject to being kept on pay at 2020 salary levels [39] until the beginning of 2023. Although on January 1, 2023, staff categories assimilated to administrative staff benefited from salary increases of 10%, these increases appeared insufficient against the background of still-high inflation [40] and salary inequities generated over time for this category of staff. Given that doctors and nurses are more professionally fulfilled and experienced significantly higher organizational justice, while administrative staff experienced significantly more EE and D, it seems that this finding is congruent with those of Maslach et al. [2] who discovered that a feeling of inequity can eventually determine professional burnout, inequity often being felt against the background of workload [34] or financial considerations [2].

Organizational justice involves a fair allocation of duties as well as strategies and methods for treating individuals fairly in the workplace [41]. Thus, these higher levels of EE and D among non-medical staff, as well as lower scores on the dimensions of personal accomplishment and organizational justice, have consequences for the wellbeing of non-medical staff.

When moderators and mediators intervene, the moderated mediation model through burnout, lifelong learning, and organizational justice in the relationship between social support and wellbeing revealed that social support has a direct effect on emotional exhaustion, lifelong learning, and wellbeing. In this study, social support was examined from the perspective of family, friends, and significant others. Thus, a high level of social support corresponds to a low level of emotional exhaustion. These results are consistent with the literature, which has shown that social support from family and community can reduce the risk of burnout by increasing resilience [42,43]. High levels of social support also correspond to high levels of wellbeing and lifelong learning. Lifelong learning is extremely valuable in medical practice and has been described as an indicator of competence and professionalism [20]. Thus, through lifelong learning, there are ways to enhance and stimulate lifelong learning. Therefore, if the individual has support from family, friends, and important people in their lives, the desire to keep up with new developments in the field increases.

Additionally, a high level of social support leads to a low level of EE. Social support can be an important resource in the workplace because of its dampening effect. When stressful events have a negative effect on a person’s wellbeing, the presence of social support can serve as a protective factor. Social support can help a person minimize perceived stress or adopt healthy behaviours in response to stressors [24,25,26]. The indirect effect of social support through emotional exhaustion, interacting with organizational justice, is significant for low, medium, or high values of the moderator, suggesting that high social support corresponds to diminished emotional exhaustion, a relationship that is moderated by organizational justice and affects wellbeing.

For example, a healthcare specialist who is complaining about workload, the lack of organizational justice, dealing with stressful situations, or emotional strain might feel overwhelmed without the social support of their loved ones and may be unable to cope with the demands of their job. Hence, if they have a strong network of friends or family members who provide understanding, empathy, and encouragement, social support can act as a defense against emotional exhaustion. This social support can provide a safe space for the individual to express their feelings, receive validation, and gain a sense of relief, thus reducing emotional exhaustion. Another example could be a professional who is facing work-related stressors such as a heavy workload, long hours, and limited work–life balance. Social support from a significant other, such as providing understanding, actively listening, and offering practical assistance, can enhance the individual’s wellbeing. By having a partner who is supportive and encourages self-care, the individual can experience reduced stress, improved work–life balance, and overall enhanced wellbeing.

Social support has a direct effect on personal accomplishment, meaning that a high level of social support corresponds to a high level of personal accomplishment. In previous studies, it was found that, in the case of healthcare professionals, social support mediates the negative effects of burnout on health, both for men and women [44]. The results of a study that analysed the impact of burnout on quality of life highlighted a positive effect of social support for healthcare assistants on the level of burnout experienced [45]. The indirect effect of social support, through personal accomplishment, interacting with organizational justice is also positive for low, high, and medium values of organizational justice, indicating that high social support corresponds to high personal accomplishment, a relationship that, in turn, is moderated by organizational justice, explaining 26% of the variation in the wellbeing dimension.

Research focusing on the relationship between organizational justice and wellbeing suggests that perceptions of fairness can make significant contributions to employee wellbeing. The significant relationship between fairness and job satisfaction suggests that employees are likely to be satisfied with their work if they perceive that they are compensated, believe that they are treated in a respectful manner, and receive timely and accurate explanations of the processes leading to the decision related to organizational fairness [46].

Social support has a significant effect on depersonalization. Previous studies examining these dimensions on samples of healthcare workers found that stressors on burnout were significantly lower in those with high social support [47].

The interaction between depersonalization and wellbeing is also significant. In previous studies, wellbeing has been conceptualized as a multidimensional construct, involving personal and professional domains that interact to feed an underlying “wellbeing reservoir.” By engaging in meaningful activities in each domain, the reservoir is sustained, but failure to meet values will lead to depletion of the reservoir, resulting in burnout. Conversely, the progression of burnout is correlated with worsening wellbeing indices [2].

The indirect effect of social support through depersonalization moderated by organizational justice influences wellbeing. Numerous studies have shown that having a network of supportive relationships contributes to psychological wellbeing. A social support network can give a sense of belonging, banish loneliness, increase feelings of self-worth, and provide a sense of security. The social network provides access to information, advice, guidance, and other assistance that the individual needs, as it is comforting to know that you have people you can turn to in a time of need [48]. The effects of depersonalization on wellbeing are significant and negative for low, high, and medium values of the moderator, indicating that high social support corresponds to low depersonalization, which, in turn, is moderated by organizational justice, explaining 22% of the variation in wellbeing.

### 4.1. Strengths and Limitations of the Study

The study is the first one providing comparative analysis for medical and nonmedical staff in Romania.

Regarding the limitations of this study, we mention the snowball sampling method used to select participants, which could lead to biased results. Another limitation concerns the variables measured with fewer items and the use of only a few dimensions of the scales, thus focusing only on those criteria that were considered relevant for this research. Using the scales in their entirety could have resulted in a very long questionnaire, which could have limited the number of respondents.

Self-reporting represents another limitation of this study, as it could lead to overestimated correlations and misinterpretations of the items. To gain a more comprehensive overview of healthcare institutions, future studies should also focus on auxiliary staff in public and private hospitals in Romania, a category that was not analysed in this research, to verify whether the proposed intervention model would yield different results for this group of employees.

To reflect reality as concisely as possible, there is a need for longitudinal research to explore the trajectory of factors that impact the perception of burnout and wellbeing among healthcare employees at different times.

This research measured the subjective perception of social support; however, it did not investigate how objective support measures taken by organizations could influence burnout and wellbeing among healthcare workers. Including objective support measures in future studies could be useful in clarifying the effects of organizational support and perceived support and would have greater practical implications for providing support measures.

### 4.2. Future Research Directions and Practical Recommendations

According to the present results, the core variables of the present research are social support, organizational justice, and wellbeing. We recommend that future studies analyse other variables that could reflect strong correlations with burnout, such as emotional intelligence as a protective factor against burnout and role conflict or role ambiguity, which could be strong predictors of burnout. Future studies could aim to extend the results by using control variables based on previous results from the literature, such as age or work experience.

Clinical implications for reducing burnout are commonly categorized as person-oriented, organization-oriented, or a combination of both [49]. Person-oriented interventions focus on strengthening individual coping strategies in the face of stress, whereas organization-oriented interventions tend to focus on reducing work demands. Recent reviews suggest that person-oriented interventions to reduce burnout are effective in the short term (6 months or less), while interventions that include both person-oriented and organization-oriented techniques are more effective in the long term [49]. The results provide information that could guide the selection of skills and techniques that could be included in person- and organization-oriented interventions to reduce burnout. For example, person-oriented interventions could include strategies for gaining social support outside the workplace, while organization-oriented interventions could focus on increasing organizational justice. In addition, staff members could benefit from learning how to bring new social contacts into their network and/or accept social support from those who provide it, as well as techniques for seeking support outside the workplace from family and friends.

Given the results of the present study, a combination of both individual and organizational interventions may have a significant impact in reducing burnout scores among doctors, nurses, physiotherapists, pharmacists, biologists, social workers, and administrative staff. Therefore, multidisciplinary actions that include changes in work environment factors along with stress management programs that teach individuals to cope more easily with stressful events have shown promising solutions to managing burnout.

Because social support affects emotional exhaustion and these factors together affect wellbeing, through organizational justice, individuals should adopt a wide range of actions that decrease emotional exhaustion and increase wellbeing. From an organizational perspective, organizational justice can be seen as an important step in reducing emotional exhaustion and promoting wellbeing.

Previous intervention models have suggested that job demands are associated with exhaustion (emotional exhaustion) and that creating optimal job demands could be the key to reducing emotional exhaustion. In addition, hospitals should recruit sufficient human resources to allow each employee to avoid suffering from excessive workload and high levels of work pressure [25]. From an organizational perspective, organizational climate and culture should focus on promoting organizational justice, which would promote support and a fair environment for all categories of personnel. In these conditions, emotional exhaustion, which recorded high values for non-medical personnel and moderate values for medical personnel in the present study, would be reduced, and wellbeing would be promoted. There are a number of strategies that organizations can adopt to promote fairness, such as equity (rewarding employees based on their contributions) and equality (maintaining reasonable levels of parity between employees) when distributing resources such as promotions, bonuses, or new job roles, with these considerations being possible especially in private institutions [50]. Other methods take the form of decisional transparency, which can include providing all employees involved in a particular decision with accurate information about decision-making processes, providing employees with explanations about why decision outcomes may have been delayed, and conducting these processes in a friendly manner that respects employees [51].

Organizational justice and social support are important factors in preventing or reducing the risk of burnout in medical and administrative personnel in hospitals. Therefore, improving these aspects could contribute to increasing the wellbeing of employees in the healthcare system. Thus, at the organizational level, fair treatment of employees or equitable distribution of tasks could reduce feelings of emotional exhaustion. Additionally, at the national level, reducing wage inequalities between administrative and medical personnel could generate a higher level of organizational justice.

From an individual perspective, certain personality traits, such as emotional stability, conscientiousness, or agreeableness, have been suggested to reduce the perception that work environments are unfavourable [52]. A protective factor against burnout has been found to be social support from family, friends, and significant others in an individual’s life. In these conditions, from an individual perspective, we can mention spending leisure time with family and friends and a balanced lifestyle between family and work. Since social support also has effects on an individual’s wellbeing and leads to lower scores of emotional exhaustion, personal initiatives to obtain social support are also indicated. A high level of social support would have consequences in the positive dimension of burnout—personal accomplishment. In particular, family support for raising children [25] or taking over household responsibilities by other family members could have direct effects on increasing the level of social support felt by medical personnel. Against the background of nursing shifts or doctor’s shifts, external organizational social support emerges as a real help.

Burnout is a problem of the whole healthcare organization rather than of certain individuals. The quality of the work organization was associated with workers’ physical and mental health. Programs that adopted organization-directed changes obtained small benefits in terms of burnout rates among physicians. Hence, work hour reductions were associated with a decrease in emotional exhaustion and depersonalization but had no effect on mean personal accomplishment [53]. Frequent studies have corroborated the presence of long-standing issues within hospital organizations, which have been extensively analysed over time. Despite multiple proposed solutions, these problems proved to be resistant to change. Certain goals, such as promoting transparency and fairness in medical appointment allocation, enhancing digital literacy, and addressing the digital gap, as well as enforcing gender equity, have emerged as common and achievable objectives that can contribute to improving the perception of organizational justice among healthcare workers. It is fundamental for healthcare managers to recognize the importance of developing employees’ perceptions of equity, as it has implications for both their wellbeing and productivity [54].

Understanding the relationship between burnout, wellbeing, social support, organizational justice, and lifelong learning is essential for healthcare professionals in Romania for distinct reasons [55,56]. Firstly, burnout is a significant issue in the healthcare field, leading to physical and emotional exhaustion, reduced personal accomplishment, and decreased quality of patient care. By understanding the factors that contribute to burnout, such as lack of social support, organizational justice, and lifelong learning, healthcare professionals can take proactive measures to prevent and manage burnout, eventually enhancing their own wellbeing and personal accomplishment. Secondly, wellbeing plays an essential role in the effectiveness and longevity of healthcare professionals’ careers. Promoting wellbeing among healthcare professionals in Romania is needed to ensure their mental, emotional, and physical health. By understanding the factors that contribute to wellbeing, such as work–life balance, self-care practices, and job resources, healthcare professionals can cultivate a supportive and healthy work environment. Thirdly, social support is crucial for healthcare professionals facing the challenges of their profession. Strong social support from family, friends, and significant others provides emotional validation, a sense of belonging, and develops coping mechanisms during stressful times. Recognizing the importance of social support and promoting supportive networks can mitigate the negative effects of burnout among healthcare professionals in Romania. Furthermore, organizational justice, which involves fair treatment, equitable practices, and transparent decision-making, is fundamental for healthcare professionals’ personal accomplishment. By promoting organizational justice, healthcare institutions in Romania can create a work environment that values and supports its employees, leading to enhanced personal accomplishment and decreased burnout rates. Lastly, lifelong learning is essential for healthcare professionals to adapt to advancements in medical knowledge and technology. Regarding non-medical personnel, digitalizing or legislative changes are essential to being up-to-date in the administrative field. Understanding the importance of continuous learning and professional development allows healthcare professionals in Romania to stay updated with the latest practices, improve their clinical skills, and deliver optimal care to patients. Lifelong learning also promotes a sense of competence and personal growth, which can contribute to personal accomplishment and wellbeing. In conclusion, comprehending the relationship between burnout, wellbeing, social support, organizational justice, and lifelong learning is critical for healthcare professionals in Romania. This understanding enables them to implement strategies that promote their own wellbeing, mitigate burnout, cultivate supportive work life balance, and continually enhance their professional skills, ultimately leading to improved healthcare outcomes.

## 5. Conclusions

We found a significantly higher level of organizational justice, personal accomplishment, and wellbeing for medical staff, while higher levels of depersonalization and emotional exhaustion were identified for non-medical staff.

When mediators and moderators intervene, social support has a direct effect on emotional exhaustion, lifelong learning, and wellbeing. Therefore, a high level of social support corresponds to a low level of emotional exhaustion. Additionally, high social support corresponds to higher values of lifelong learning and wellbeing. Organizational justice was a viable moderator of the relationship between emotional exhaustion and wellbeing. As expected, social support has a significant positive effect on personal accomplishment. Additionally, a significant effect of personal accomplishment on wellbeing could be found, as well as for the interaction between organizational justice and personal accomplishment. The indirect effect of social support, through personal accomplishment, on interaction with organizational justice, is also positive. Social support also has a significant effect on depersonalization, and the interaction between depersonalization and wellbeing is also significant. The indirect effect of social support through depersonalization, moderated by organizational justice, is significant, as is the index of moderated mediation. Based on these results, the intervention models aim for a combined approach between the organizational and the personal levels of the individual. They aim to increase social support from family, friends, and significant others, which has the effect of reducing emotional exhaustion and promoting wellbeing.

## Figures and Tables

**Table 1:** Socio-demographic, professional, and institution-related data * (*n* = 497).

Characteristics	*n*	%
**Gender**		
Male	74	14.89%
Female	423	85.11%
**Marital status**		
Single	98	19.72%
In a relationship	399	80.28%
**Being parents**		
Yes	377	75.86%
**Professional category**		
Nurse	216	43.46%
Administrative personnel	130	26.16%
Physician	97	19.52%
Other medical personnel	54	10.86%
**Job tenure in years**		
˂5 years	89	17.91%
6–10 years	82	16.50%
11–20 years	120	24.14%
>20 years	206	41.45%
**Type of organization**		
Public	360	72.43%
Private	62	12.48%
Public and private	75	15.09%
**Emergency unit**		
Yes	174	35.01%
**Team coordinator**		
Yes	122	24.55%
**Education**		
High school/post-secondary studies	141	28.37%
Certificate or qualification diploma	8	1.61%
Bachelors’ degree	187	37.63%
Master’s degree	131	26.36%
Ph.D. studies/Post doc	30	6.03%
**Chronic disease**		
Yes	147	29.58%

* *n* (number of subjects) and % (percentages).

**Table 2:** Descriptive statistics and Pearson correlations *.

	Variables	M (SD)	1	2	3	4	5	6	7	8
**Medical**	1. Emotional exhaustion	19.67 (0.71)	-							
	2. Personal accomplishment	38.66 (0.39)	−0.27 **	-						
	3.Depersonalization	4.43 (0.26)	0.50 **	−0.33 **	-					
	4. Organizational justice	24.28 (0.24)	−0.36 **	0.21 **	−0.23 **	-				
	5. Lifelong learning	45.66 (0.31)	−0.13 *	0.29 **	−0.11 *	0.13 **	-			
	6. Wellbeing	11.95 (0.21)	−0.54 **	0.39 **	−0.34 **	0.26 **	0.21 **	-		
	7. Social Support	5.83 (0.04)	−0.22 **	0.29 **	−0.20 **	0.25 **	0.25 **	0.33 **	-	
	8. Age	43.75 (0.50)	0.03	0.14 **	−0.10 *	−0.12 *	0.10 *	0.03	−0.07	-
	9. Job tenure	12.97 (0.54)	0.08	0.07	−0.08	−0.15 **	0.05	−0.02	−0.02	0.65 **
**Nonmedical**	1. Emotional exhaustion	27.33 (1.24)	-							
	2. Personal accomplishment	35.87 (0.69)	−0.11	-						
	3. Depersonalization	6.59 (0.52)	0.39 **	−0.15	-					
	4. Organizational justice	22.14 (0.38)	−0.19 *	0.17 *	−0.09	-				
	5. Lifelong learning	45.03 (0.52)	0.10	0.37 **	0.05	0.07	-			
	6. Wellbeing	10.33 (0.37)	−0.53 **	0.35 **	−0.17 *	0.15	0.04	-		
	7. Social support	5.76 (0.09)	−0.04	0.20 *	0.01	0.15	0.06	0.33 **	-	
	8. Age	45.63 (0.80)	0.16	0.05	−0.16	−0.06	0.23 **	−0.08	−0.15	-
	9. Job tenure	11.39 (0.85)	0.24 **	0.00	0.00	0.11	−0.01	−0.24 **	−0.13	0.41 **

* *p* < 0.05; ** *p* < 0.01.

**Table 3:** T test for independent sample.

Variables	Medical Personnel	Non-Medical Personnel	t	Cohen’s d
Emotional exhaustion	19.67 ± 0.71	27.33 ±1.24	−5.43 **	−0.55
Personal accomplishment	38.66 ± 0.39	35.87 ± 0.69	3.59 **	0.36
Depersonalization	4.43 ± 0.26	6.59 ± 0.52	−3.99 **	−0.40
Organizational justice	24.28 ± 0.24	22.14 ± 0.38	5.57 **	0.56
Lifelong learning	45.66 ± 0.31	45.03 ± 0.52	1.02	0.10
Wellbeing	11.95 ± 0.21	10.33 ± 0.37	3.81 **	0.38
Social support	5.83 ± 0.04	5.76 ± 0.09	0.72	0.07

** *p* < 0.01.

## Data Availability

Data are available upon request from the corresponding author.
